# Dipeptidyl peptidase-1 inhibition with brensocatib reduces the activity of all major neutrophil serine proteases in patients with bronchiectasis: results from the WILLOW trial

**DOI:** 10.1186/s12931-023-02444-z

**Published:** 2023-05-17

**Authors:** David Cipolla, Jimin Zhang, Brice Korkmaz, James D. Chalmers, Jessica Basso, Daniel Lasala, Carlos Fernandez, Ariel Teper, Kevin C. Mange, Walter R. Perkins, Eugene J. Sullivan

**Affiliations:** 1 Insmed Incorporated, 700 US Highway 202/206, Bridgewater, NJ 08807 USA; 2 INSERM UMR-1100, “Research Center for Respiratory Diseases” and University of Tours, Tours, France; 3 Division of Molecular and Clinical Medicine, University of Dundee, Ninewells Hospital and Medical School, Dundee, UK

**Keywords:** Non-cystic fibrosis bronchiectasis, Brensocatib, Dipeptidyl peptidase-1 inhibitor, Neutrophil serine protease, Sputum biomarkers, Neutrophil elastase, Proteinase 3, Cathepsin G

## Abstract

**Background:**

Brensocatib is an oral, selective, reversible inhibitor of dipeptidyl peptidase-1 (DPP-1), responsible for activating neutrophil serine proteases (NSPs) including neutrophil elastase (NE), proteinase 3 (PR3), and cathepsin G (CatG). In chronic inflammatory lung diseases such as non-cystic fibrosis bronchiectasis (NCFBE), neutrophils accumulate in the airways resulting in excess active NSPs that cause damaging inflammation and lung destruction.

**Methods:**

The 24-week WILLOW trial (NCT03218917) was a randomized, double-blind, placebo-controlled, parallel-group trial in patients with NCFBE conducted at 116 sites across 14 countries. In this trial, treatment with brensocatib was associated with improvements in clinical outcomes including time to first exacerbation, reduction in exacerbation frequency and a reduction in NE activity in sputum. An exploratory analysis of NE activity in white blood cell (WBC) extracts and NE, PR3 and CatG activity in sputum was conducted to further characterize brensocatib’s effect and identify potential correlated effects.

**Results:**

NE, PR3 and CatG activities were reduced in sputum and NE activity was reduced in WBC extracts in a dose-dependent manner after four weeks of brensocatib treatment, with a return to baseline four weeks after the end of treatment. Brensocatib produced the greatest reduction in the sputum activity of CatG, followed by NE and then PR3. Positive correlations among the sputum NSPs were observed both at baseline and in response to treatment, with the strongest correlation among the sputum NSPs for NE and CatG.

**Conclusions:**

These results suggest a broad anti-inflammatory effect of brensocatib underlying its clinical efficacy observed in NCFBE patients.

*Trial registration:* The study was approved by the corresponding ethical review boards of all participating centers. The trial was approved by the Food and Drug Administration and registered at clinicaltrials.gov (NCT03218917) on July 17, 2017 and approved by the European Medicines Agency and registered at the European Union Clinical trials Register (EudraCT No. 2017-002533-32). An independent, external data and safety monitoring committee (comprising physicians with pulmonary expertise, a statistician experienced in the evaluation of clinical safety, and experts in periodontal disease and dermatology) reviewed all adverse events.

**Supplementary Information:**

The online version contains supplementary material available at 10.1186/s12931-023-02444-z.

## Background

Non-cystic fibrosis bronchiectasis (NCFBE) is a chronic, progressive respiratory disease defined by permanent dilatation of the bronchi [1]. Pathogenesis in NCFBE is complex, involving not only inflammatory components and lung tissue damage, but also infection and airway dysfunction [2–4]. These four features have been termed the vicious cycle [1] or more recently, the vicious vortex [3]. Neutrophils accumulate in the airways resulting in excess neutrophil serine protease (NSP) activity that can lead to destruction of lung tissue [2–4]. The three main NSPs include neutrophil elastase (NE), proteinase 3 (PR3), and cathepsin G (CatG). Elevations in NE activity in sputum in NCFBE are associated with increased exacerbations and declines in lung function [5]. Excess PR3 and CatG activity may also play a role in NCFBE disease progression [6, 7]. Thus, reduction of sputum NSP activity, as opposed to inhibition of NE exclusively, represents a novel therapeutic approach to attenuate this vicious vortex in NCFBE [2, 3].

Dipeptidyl peptidase-1 (DPP-1, EC 3.4.14.1), also known as cathepsin C, is an enzyme responsible for activating NE, PR3, and CatG zymogens in promyelocytes during neutrophil differentiation in the bone marrow [8]. Collectively these proteolytic enzymes are stored within neutrophil granules and released during degranulation and neutrophil extracellular trap release (NETosis) [9]. Human loss-of-function mutations in DPP-1 in Papillon-Lefèvre syndrome subjects markedly reduce NSP protein and proteolytic activities (active NE/PR3 < 5%; active CatG < 1%) in mature blood neutrophils [10], demonstrating that DPP-1 is a key regulator of NSP activation in humans [8]. Importantly, patients with Papillon-Lefèvre syndrome are not typically at increased risk for infections [8].

Brensocatib is an investigational, oral, selective, reversible inhibitor of DPP-1 [11, 12]. Evaluation of once-daily brensocatib (10, 25 or 40 mg) in healthy subjects for 21 or 28 days resulted in a dose-dependent reduction in NE activity in neutrophils in the blood, achieving maximal reductions of 30, 49 and 59%, respectively, after 28 days [12]. The one-month time frame to achieve maximal and steady-state effect on blood NE levels is consistent with a 15-day maturation time for human neutrophils in the bone marrow. This phase 1 study did not report effects of brensocatib on PR3 and CatG but established the potential for DPP-1 inhibitors like brensocatib to attenuate NSP activity in neutrophil-driven inflammatory diseases.

In a phase 2b, randomized, double-blind, placebo-controlled trial in 256 patients with NCFBE (WILLOW), 24 weeks of treatment with 10 or 25 mg brensocatib was associated with improvements in clinical outcomes including prolongation in the time to first exacerbation and reduction in the frequency of exacerbations [11]. Of the 256 patients, 87 were assigned to receive placebo, 82 to receive 10 mg of brensocatib, and 87 to receive 25 mg of brensocatib. Additionally, brensocatib reduced NE activity in sputum reaching a nadir at the 4-week timepoint that was maintained throughout the 24-week treatment duration, with a return to baseline levels at the 28-week timepoint, four weeks after termination of brensocatib treatment [13]. The achievement of sputum NE activity below the limit of quantitation (BLQ) post baseline was also associated with a statistically significant reduction in pulmonary exacerbations [14].

In this manuscript, we conducted an exploratory analysis to evaluate the effect of brensocatib on PR3 and CatG activity in sputum, and NE activity in white blood cell (WBC) extracts, which were not previously reported in the phase 2b trial in NCFBE [13]. We also compared the extent of reduction of NSP activity in both WBC extracts and sputum compartments and identified correlations in all three NSP activities at baseline and on treatment. These data may inform on the effect of a reversible DPP-1 inhibitor like brensocatib on the relevant pharmacodynamic (PD) biomarkers and their contribution to morbidity in NCFBE.

## Methods

### Trial design and data analysis

The WILLOW trial (ClinicalTrials.gov: NCT03218917; EudraCT: 2017-002533-32) was a randomized, double-blind, placebo-controlled, parallel-group trial conducted at 116 sites across 14 countries [13]. According to the study protocol, eligible patients were 18 to 85 years of age and had a clinical history consistent with bronchiectasis (i.e., cough, chronic sputum production, or recurrent respiratory infections), as confirmed on computed tomography of the chest. Patients had to have at least two documented exacerbations in the previous 12 months, a history of chronic sputum expectoration, sputum color at screening that was rated as being mucopurulent or purulent according to a validated color chart [13], and the ability to provide a sputum sample during screening.

Key exclusion criteria included bronchiectasis due to a clinical diagnosis of cystic fibrosis, hypogammaglobulinemia, common variable immunodeficiency, or alpha1-antitrypsin deficiency, as well as an investigator-determined primary diagnosis of chronic obstructive pulmonary disease or asthma (secondary diagnoses were allowed). Because of potential dental side effects of treatment with brensocatib, exclusion criteria were structured to avoid the enrollment of patients with severe periodontitis.

The WILLOW Statistical Analysis Plan (SAP) is discussed in the Additional file 1. The effect of brensocatib compared to placebo on activity of NE in WBC extracts and PR3 and CatG in sputum were exploratory objectives, as were the correlations of NE, PR3 and CatG activities in sputum. Details of the data analysis criteria for the WBC extracts and sputum NSP data sets are described in the Additional file 1.

### WBC collection procedure

Blood was collected from participating patients at each of the following visits: Day 1 (pre-dose), weeks 2, 4, 12, 24 (the end of the treatment period), and at week 28 (4 weeks after the end of the treatment period) [13]. A total of 2 mL whole blood was obtained from each patient into the citrated blood tubes and inverted 4 times. The whole blood samples were processed into WBC pellets by lysing 2 mL whole blood with 40 mL 1 × lysis buffer (Abcam, Cat No. Ab204733), inverting 5 times, and incubating at room temperature for 20 min [15]. Samples in 50 mL falcon tubes were then centrifuged (400×*g* for 5 min, 4 °C), followed by carefully decanting the liquid without disrupting the WBC pellet. The retained WBC pellets were frozen and stored at − 80 °C prior to subsequent NSP extraction.

### NE extraction from WBC pellets

An NSP extraction methodology utilizing surfactant and mechanical agitation has previously been described and qualified [15]. Briefly, individual WBC pellet samples were thawed at room temperature. Following thawing, all steps of this procedure were performed on ice. All WBC pellet samples were lysed via mechanical pipetting of each sample with 1 mL lysis buffer comprising Nonidet P40 Substitute (Sigma, Cat No. 98379). Post lysis, samples were centrifuged (16,000×*g* for 10 min, 4 °C) to pellet the cell debris. A total of three lysis cycles was performed on each pellet, with lysate fractions being pooled after each cycle. The pooled lysis supernatants were then aliquoted and stored at − 80 °C until NSP activity quantitation.

### Quantitation of WBC NE activities

To quantify the activity of NE in the WBC pellets, kinetic enzymatic assays were applied to the WBC lysate samples as previously described [15]. Briefly, these enzymatic assays used fluorogenic substrates specifically cleaved by active NE to generate fluorescent 7-amino 4-methyl coumarin (AMC). Increases in the activity of NE produced increased amounts of fluorescence in the assays. This assay does not possess a lower limit of quantitation for NE activity in the WBC pellets.

For the data analyses, only patients with baseline WBC extract NE values were evaluated to enable a determination of the change in NE activity from baseline. There were patients with missing WBC extract NE values at baseline (and at subsequent time points) due to misapplication of the WBC pellet procedure by the clinical sites leading to samples becoming compromised and unable to be assayed.

### Sputum collection procedure

Sputum was collected from participating patients at each of the following visits: Screening Visit, Day 1 (pre-dose), weeks 2, 4, 12, 24 (the end of the treatment period), and at week 28 (4 weeks after the end of the treatment period) [13]. Patients were instructed to open the top of the sputum cup funnel and expectorate into the container until the sputum reached the 1 mL line (or more) on the plastic tube. The tube was tightly capped and immediately placed upright in a tube rack or box and frozen at − 70 °C at the central lab until ready for sputum NSP analysis.

### Quantitation of sputum NSP activity

To measure the activity of NE, PR3, and CatG in sputum, the ProAxsis ProteaseTag® Active NSP Immunoassays (ProAxsis, Belfast, UK) were used as a sandwich ELISA assay. Immunoassay plates were coated with a capture tag which allowed selective bindings to the active form of the specific NSP (NE, PR3 or CatG) on the plates. Phosphate buffered saline was added to the sputum at a 1:4 V:W ratio (volume added (mL) = mass of sample (g) × 4 mL) to homogenize the sputum samples. Sputum aliquots were then diluted with assay diluent (200-fold, 50-fold, tenfold, undiluted) and incubated in the ProAxsis plates. Then a primary antibody against each NSP was added to the plate and incubated to allow binding to the captured NSP. If the primary antibody was not conjugated, then a secondary horse radish peroxidase (HRP) conjugated antibody was added to the plate and incubated to allow binding to the primary antibody. A color forming substrate containing tetramethylbenzidine was also added to generate a colored product via an enzymatic reaction with the HRP, which was followed by an immediate absorbance reading using a Molecular Devices plate reader. Increases in NSP activity yielded increased amounts of absorbance in each of these assays.

### Sputum NSP data analysis criteria

The baseline sputum NSP values represented the average of the sputum NSP values from the subject’s Screening visit and Day 1 (pre-dose) visit as both visits were prior to the initiation of treatment. If one of the Screening or the Day 1 NSP values was below the quantification limit (BQL), then the non-BQL value was used as the Baseline value. If both values at the Screening visit and Day 1 (pre-dose) visit were BQL, then the baseline value was represented by a value of zero.

Every subject with a non-zero baseline sputum NSP value was included for further data analysis where comparison to baseline was performed. For those subjects with a non-zero baseline value, if any post-baseline value was BQL, then for each visit with a BQL value, the value of the BQL character field was converted to zero. Due to the lognormal distribution nature of the sputum NSP data set, a data transformation method using Log transformation replaced each numeric number x with log_10_(x).

### Correlation of sputum NSPs

The correlation of sputum NSP activities was determined for all patients combined, irrespective of treatment arms at baseline and for all timepoints. The correlations of sputum NSP activities were further analyzed according to treatment group: placebo, 10 mg brensocatib, or 25 mg brensocatib at baseline and for each timepoint: week 2, week 4, week 12, week 24 and week 28.

## Results

### Distribution analysis of WBC and sputum NSP activities

The WILLOW trial randomized 256 patients, and 89, 81 and 85 had sputum NSP activity data in the 25 mg brensocatib, 10 mg brensocatib, and placebo groups, respectively. Baseline and post-baseline NE activity data in blood was reportable for 37, 30 and 35 patients, respectively. The prespecified analysis of sputum NSP activities assumed that they followed a lognormal distribution profile. This assumption was confirmed for NE, PR3 and CatG. The WBC NE activity followed a normal distribution profile. These analyses are shown in Additional file 1: Figs. S1–S4.

### Effect of brensocatib treatment on active NSP levels in sputum

**Fig. 1:**
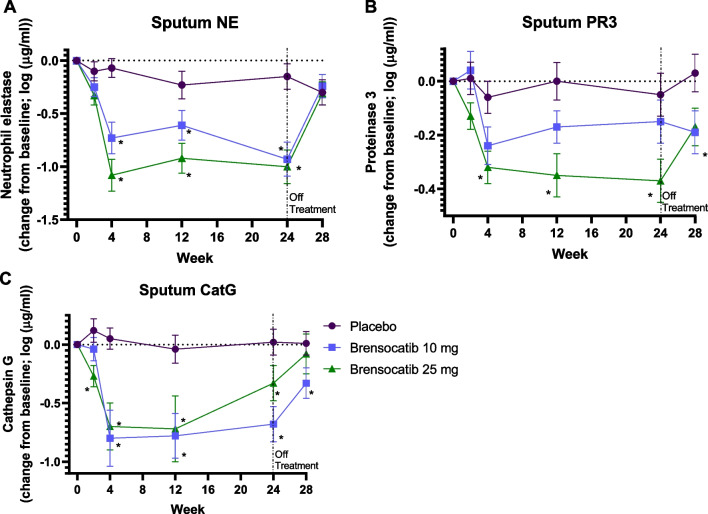
NSP activity in sputum on study. Change from baseline is presented as mean (± SEM) for (**a**) NE, (**b**) PR3 and (**c**) Cat G. *, P < 0.05 vs placebo at the same timepoint. Figure **a** is from [New England Journal of Medicine, Chalmers, J.D. Haworth, C.S.; Metersky, M.L. et al. Phase 2 Trial of the DPP-1 Inhibitor Brensocatib in Bronchiectasis, 383, 2135. Copyright © (2020) Massachusetts Medical Society. Reprinted with permission from Massachusetts Medical Society]

Active sputum NE levels were reduced by brensocatib by week 4 in a dose-dependent manner, with greater reductions for the 25 mg dose as compared to the 10 mg dose (Fig. 1). NE activities remained reduced throughout the 24-week course of treatment, then started to recover 4 weeks after the end of treatment. The mean NE activity at week 4 was 1514 ng/mL for placebo, 214 ng/mL for 10 mg brensocatib, and 141 ng/mL for 25 mg brensocatib, representing a reduction in NE activity compared to placebo of 86% and 91% for 10 and 25 mg brensocatib, respectively (Table 1). A similar trend was observed for CatG (Fig. 1). The mean CatG activity at week 4 was 56 ng/mL for placebo, 6 ng/mL for 10 mg brensocatib, and 4 ng/mL for 25 mg brensocatib, representing a reduction in CatG activity compared to placebo of 90% and 93% for 10 and 25 mg brensocatib treatment, respectively (Table 1). PR3 activity was reduced to a lesser extent than for NE and CatG, but also showed a dose dependent response (Fig. 1). The mean PR3 activity was 2927 ng/mL for placebo, 2309 ng/mL for 10 mg brensocatib, and 1368 ng/mL for 25 mg brensocatib, representing a reduction in PR3 activity relative to placebo of 21% and 53% for 10 and 25 mg brensocatib treatment, respectively (Table 1).

**Table 1:** Reduction in active NSPs in sputum and in WBCs at Week 4 relative to placebo

NSP	Brensocatib	Week	WBCs^a^	Sputum^b^
NE	10 mg	4	− 20%	− 86%
	25 mg	4	− 62%	− 91%
PR3	10 mg	4	NA	− 21%
	25 mg	4	NA	− 53%
CatG	10 mg	4	NA	− 90%
	25 mg	4	NA	− 93%

The mean NSP activity at week 4 for the 10 and 25 mg brensocatib groups was compared to that for the placebo group at week 4 to determine a percent reduction relative to placebo. *NA* not applicable

^a^Reduction in arithmetic mean

^b^Reduction in geometric mean

### Effect of brensocatib treatment on active NE levels in WBCs

**Fig. 2:**
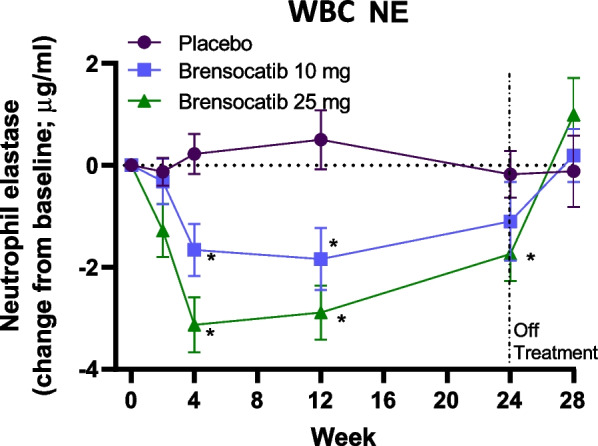
NE activity in WBCs on study. Change from baseline is presented as mean (± SEM) for NE. * P < 0.05 vs placebo at the same timepoint

The total number of reportable NE activities from the blood samples at baseline and over the course of the trial was 256 for placebo, 239 for 10 mg brensocatib, and 299 for 25 mg brensocatib. The effect of treatment on NE activity in WBCs was also evaluated. Active NE levels in WBC extracts were reduced by brensocatib by week 4 in a dose-dependent manner, which was maintained over the 24 weeks, with a recovery to baseline levels 4 weeks after the end of treatment (Fig. 2). After 4 weeks of treatment, mean NE activity was 5.77 ng/mL for placebo, 4.59 ng/mL for 10 mg brensocatib, and 2.17 ng/mL for 25 mg brensocatib, representing a reduction in NE activity compared to placebo of 20% and 62%, for 10 and 25 mg brensocatib, respectively (Table 1). While similar trends were observed in WBCs and sputum NE activity, both in the timing and duration of effects, NE activity in sputum was reduced to a greater extent than in WBCs.

### Correlation of effect on active sputum NSP levels

**Fig. 3:**
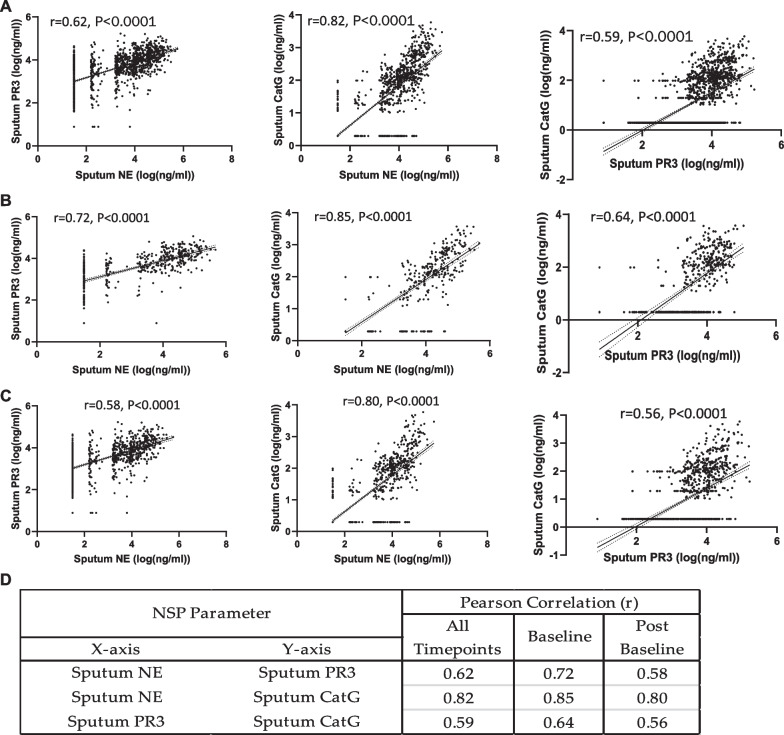
Correlation of sputum NSP activities from all treatment arms. Pearson correlation analysis within sputum NSPs at (**a**) all timepoints, (**b**) at baseline and (**c**) at only post-baseline time points. (**d**) Summaries of the Pearson correlation coefficients (r) between each sputum NSP activity from baseline values and from values at all timepoints. Simple linear regression was plotted with the best-fit line plus 95% confidence intervals. The total number of paired data points from baseline through week 28 are: NE and PR3 (n = 1073), NE and CatG (n = 1085) and PR3 and CatG (n = 1070)

We were further interested in understanding any possible correlations among the sputum NSP activities. The three pairs of sputum NSP biomarkers are plotted in Fig. 3a. There were strongly positive Pearson Correlation Coefficients (r) for all three pairs of sputum NSP biomarkers. The strongest correlation (r = 0.82) was between the sputum NE and CatG activities, followed by NE and PR3 (r = 0.62) and PR3 and CatG (r = 0.59). To understand specifically if there was any effect of treatment on the NSP correlations, the analysis was repeated separately for both the baseline NSP values (Fig. 3b) and for the post-baseline NSP values (Fig. 3c). For both sets of analyses, the rank order remained the same with the strongest correlation between NE and CatG. The correlation coefficients increased slightly when the analysis was performed only for the baseline values and decreased slightly for the post-baseline values, but the range in r values remained narrow: NE and CatG correlations varied from 0.80 (post baseline) to 0.85 (baseline only), NE and PR3 varied from 0.58 (post baseline) to 0.72 (baseline only), and PR3 and CatG varied from 0.56 (post baseline) to 0.64 (baseline only). The Pearson Correlations for the paired sputum NSP activities are summarized in Fig. 3d.

**Fig. 4:**
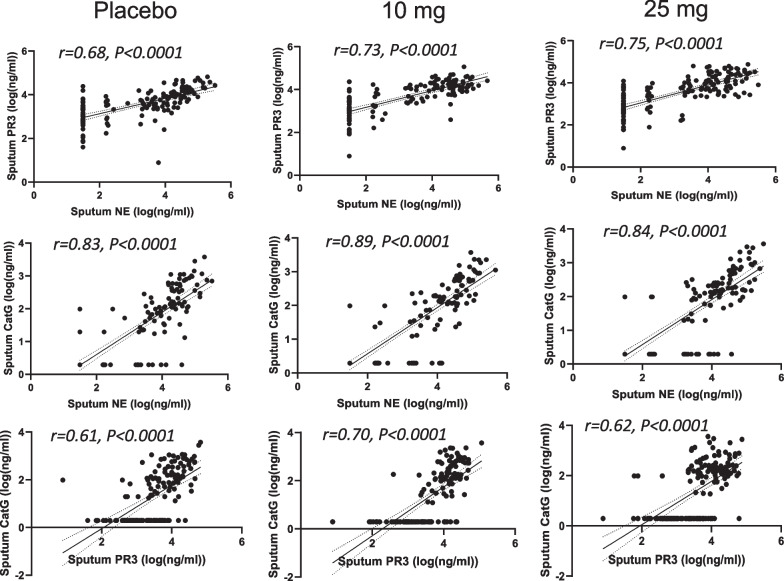
Correlation of sputum NSPs in separate dosing arms at baseline only. NSP sputum correlation analysis was performed for placebo, 10 mg brensocatib and 25 mg brensocatib. Simple linear regression was plotted with the best-fit line plus 95% confidence intervals

**Fig. 5:**
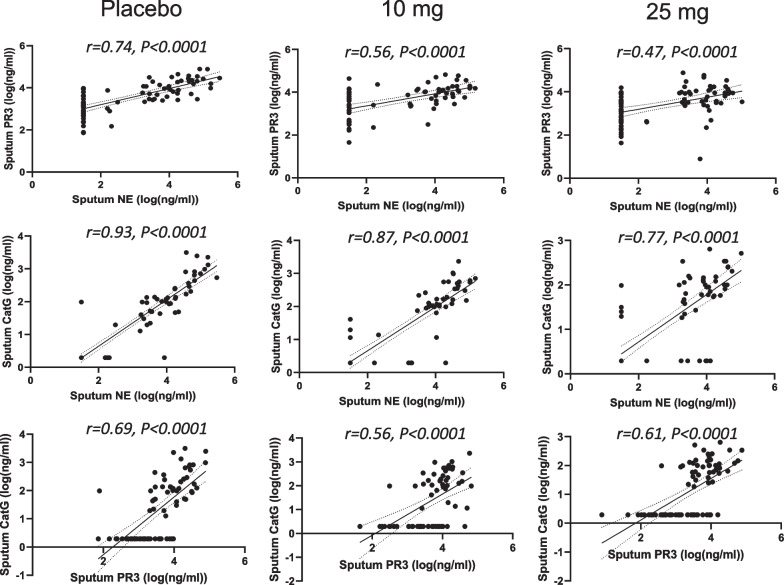
Correlation of sputum NSPs in separate dosing arms at Week 2. NSP sputum correlation analysis was performed for placebo, 10 mg brensocatib and 25 mg brensocatib. Simple linear regression was plotted with the best-fit line plus 95% confidence intervals

**Fig. 6:**
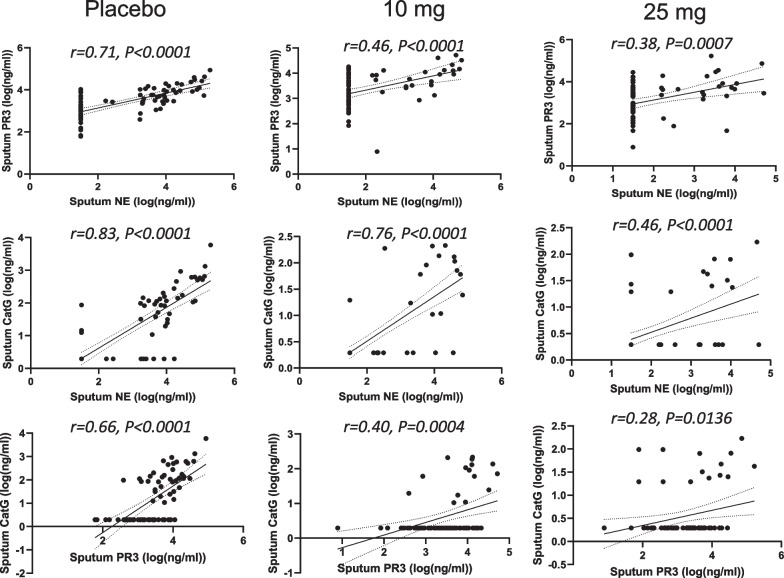
Correlation of sputum NSPs in separate dosing arms at Week 4. NSP sputum correlation analysis was performed for placebo, 10 mg brensocatib and 25 mg brensocatib. Simple linear regression was plotted with the best-fit line plus 95% confidence intervals

**Fig. 7:**
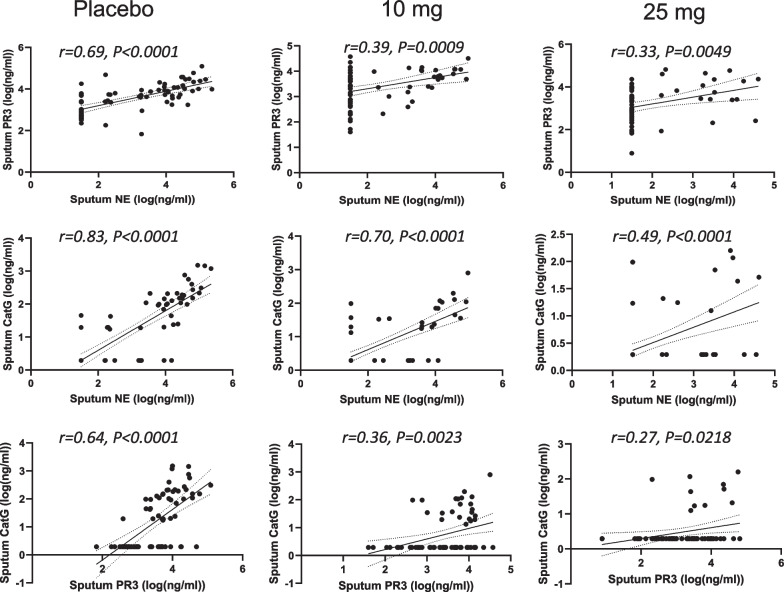
Correlation of sputum NSPs in separate dosing arms at Week 12. NSP sputum correlation analysis was performed for placebo, 10 mg brensocatib and 25 mg brensocatib. Simple linear regression was plotted with the best-fit line plus 95% confidence intervals

**Fig. 8:**
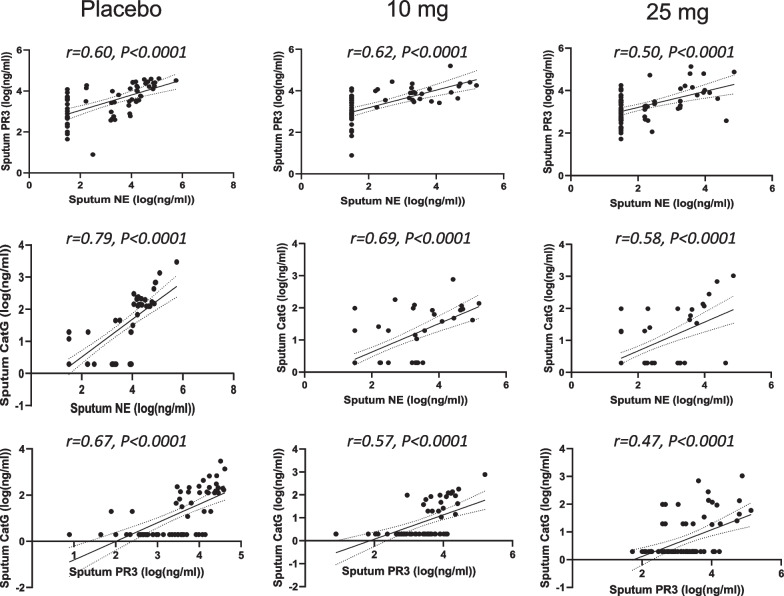
Correlation of sputum NSPs in separate dosing arms at Week 24. NSP sputum correlation analysis was performed for placebo, 10 mg brensocatib and 25 mg brensocatib. Simple linear regression was plotted with the best-fit line plus 95% confidence intervals

**Fig. 9:**
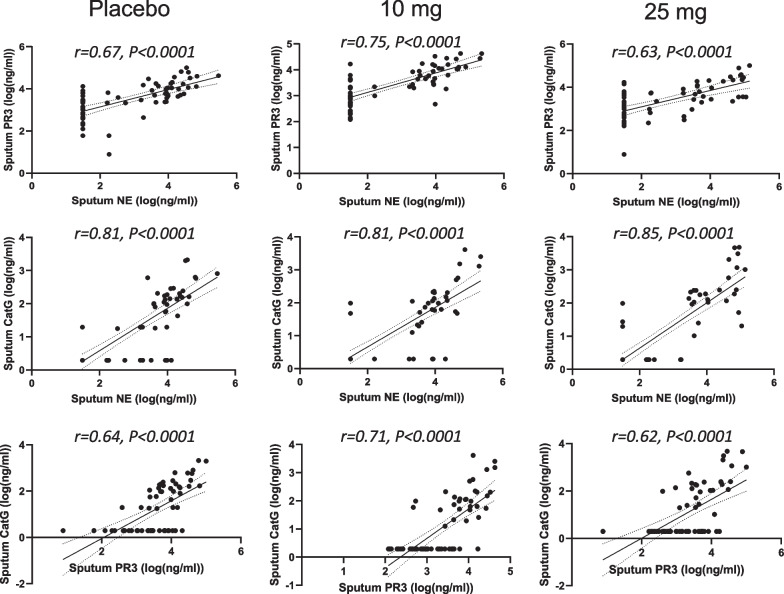
Correlation of sputum NSPs in separate dosing arms at Week 28. NSP sputum correlation analysis was performed for placebo, 10 mg brensocatib and 25 mg brensocatib. Simple linear regression was plotted with the best-fit line plus 95% confidence intervals

**Fig. 10:**
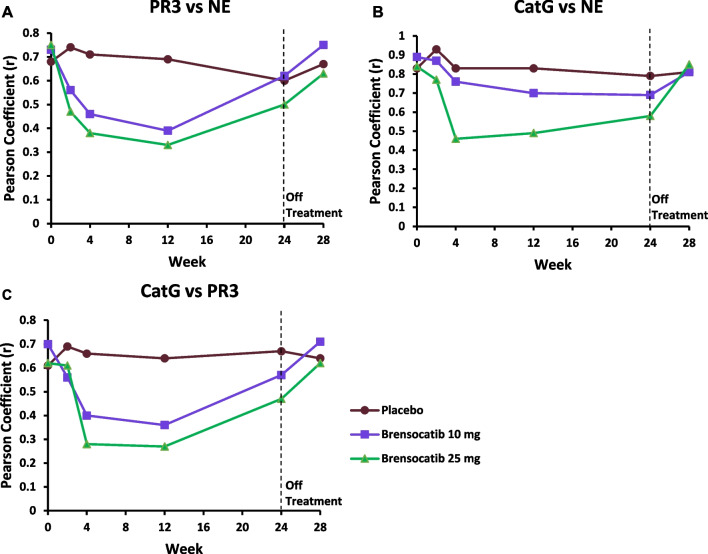
Pearson correlation coefficients (r) for each pair of sputum NSP activities over the trial duration. Pearson correlation coefficients (r) are presented as mean for (**a**) PR3 vs NE, (**b**) CatG vs NE and (**c**) Cat G vs PR3

The slightly less positive NSP correlation values post baseline suggested that there may have been an effect of treatment. Thus, the correlation analysis was repeated for each of the three dosing groups to identify if the NSP correlations for patients on brensocatib differed compared to those on placebo over the course of the trial: at baseline (Fig. 4), week 2 (Fig. 5), week 4 (Fig. 6), week 12 (Fig. 7), week 24 (Fig. 8) and week 28 (Fig. 9). The NSP correlations are plotted for each treatment group over the duration of the trial (Fig. 10). The last post-baseline timepoint occurred at week 28, 4 weeks after completion of therapy and the NSP correlations returned to baseline levels.

## Discussion

Bronchiectasis is an inflammatory disorder and neutrophils dominate the airway inflammatory profile in the majority of bronchiectasis patients. NSPs such as NE, PR3 and CatG are released from neutrophil granules when neutrophils are activated and during the formation of neutrophil extracellular traps [1, 9]. The resulting protease damage to the airways has been linked to impaired host defense through cleavage of host receptors and antimicrobial peptides leading to increased susceptibility to infection and disease progression through extracellular matrix degradation. Thus, elevated NE activity has been shown to predict risk of exacerbation, lung function decline over time and mortality in bronchiectasis [5]. The association between CatG and PR3 sputum activity and outcomes in bronchiectasis has not been thoroughly investigated. However, in CF patients, PR3 and CatG are found at high concentrations in the sputum and bronchial alveolar lavage fluid so they should not be discounted as contributors to lung disease in NCFBE [6]. This study shows strong correlations among NE, CatG and PR3 activities in sputum suggesting the potential for elevated CatG and PR3 activity to be associated with increased exacerbation risk.

There are currently no approved treatments for bronchiectasis, and there is a need for non-antibiotic treatments that can reduce lung inflammation. To that end, inhibition of DPP-1 represents a novel therapeutic strategy to reduce NSP activity and lung inflammation [13]. While brensocatib’s direct mechanism of action is to reduce active NSPs in circulating neutrophils, the pharmacologic effect in bronchiectasis relies on a reduction of NSP activity in the lung and these reductions were indeed observed in sputum samples. NSP activity in sputum is ultimately a consequence of circulating blood neutrophils migrating to the lung and releasing their NSP cargo. Thus, it is not unexpected that the reduction in circulating neutrophil NSP activity directly translates to reduced NSP activity in the lung.

Interestingly, a greater reduction of NE activity was observed in sputum than in WBCs (Table 1), suggesting that it may not be necessary to completely inhibit DPP-1 in circulating neutrophils to have a meaningful impact on lung markers of inflammation. Biomarker analysis planned for the ongoing Phase 3 brensocatib ASPEN trial (ClinicalTrials.gov Identifier: NCT04594369) and Phase 2 trial in CF (NCT05090904) may inform on whether CatG and PR3 activities are also reduced in sputum to a greater extent than in WBCs.

There are several plausible explanations for a greater effect in the lung than in the circulation. Reduced NSP activity may directly or indirectly reduce neutrophil recruitment to the lung, or the inflammatory stimulus for NSP release including potentially impacting bacterial infection through restoration of antimicrobial peptides. This theoretically generates a “virtuous cycle”, the counterpoint to the vicious cycle, whereby reductions in lung inflammation improve other aspects of airway disease, leading to larger and more sustained reductions in airway inflammation. These reductions in NSP activity in the lung may allow for a return to homeostatic balance of NSPs with their inhibitors: alpha-1 antitrypsin (A1AT), elafin and secretory leukocyte peptidase inhibitor (SLPI).

The positing of a virtuous cycle in response to brensocatib suggests that once brensocatib treatment is removed, NSP activities in the lung may take longer to return to baseline levels than for those in the circulation (i.e., WBC extracts). Indeed, four weeks after termination of brensocatib treatment, NE activity in the WBCs returned to baseline levels, while NE, PR3 and CatG activities in sputum did not.

Sputum NE, PR3 and CatG activities were strongly correlated at baseline and positive correlations were observed on study treatment at all timepoints, suggesting that brensocatib’s effect on all three NSP activities is interrelated. This does not appear to be a surprising result given that the mechanism of action of DPP-1 is to activate all three NSPs. However, while the NSP correlations remained positive on treatment, the strength of those correlations decreased somewhat for those on brensocatib, and more so for those on the higher 25 mg dose of brensocatib. The explanation may be related to the differential reduction in the NSP activities to brensocatib treatment, with CatG being reduced to the greatest extent, followed closely by NE, with a lower reduction in PR3 activity in sputum. Thus, it may not be unexpected that the very strong correlation between NE and CatG at baseline showed the least change in response to brensocatib treatment, since both biomarkers were more likely to have been reduced comparably. In contrast, the positive correlations between PR3 and both NE and CatG declined on brensocatib treatment (but not on placebo), which may be explained by the lower reduction in PR3 activity relative to both NE and CatG. The residual reductions in the NSP correlations that cannot be explained by differential impact of brensocatib on the individual NSP biomarkers may be related to the increased appearance of NSP activity values below the quantification limit. Notably, four weeks after the end of brensocatib treatment, the sputum NSP correlations all recovered close to their initial values.

DPP-1 inhibition with brensocatib had the greatest effect in reducing CatG activity in sputum and the least effect on PR3 activity. There may be multiple factors accounting for the differential response in NSP activity to DPP-1 inhibition. In the absence of a DPP-1 inhibitor, nearly all of the NSPs zymogens are converted to active forms during neutrophil maturation [16]. Studies with DPP-1 knockout mice (dpp1-/-) suggest that there is a “DPP-1-like” protease that activates a portion of NE and PR3 zymogens (app. 10%) but < 1% of CatG zymogen is converted to its active form [17]. This DPP-1-like protease termed as NSPs-Alternative Activating Protease (NSPs-AAP) is also present in humans and maturates < 5% of NE/PR3 zymogens and < 1% of CatG zymogen [10]. Thus, if NSPs-AAP is not inhibited by brensocatib, then CatG activity would be reduced to a greater extent than NE or PR3, as was observed. The greater reduction in NE activity in sputum compared to PR3 may be a consequence of differences in binding affinity of their zymogens to either DPP-1 or the NSPs-AAP, as well as the presence of protease inhibitors in the sputum like A1AT that has preferential binding affinity to NE [18]. The presence of bound A1AT to the active site of NE (or PR3 or CatG) may lead to it not being quantified in our NSP activity assay.

Brensocatib reduced all three NSP biomarkers in sputum illustrating the unique mechanism of action of DPP-1 inhibitors that simultaneously reduce several key inflammatory mediators linked to exacerbation. In contrast, specific inhibitors to NE ([19] for review, [20, 21]), while showing promising preclinical data, have failed to achieve their primary endpoints in cystic fibrosis, NCFBE or chronic obstructive pulmonary disease, even though NE is associated with worsening disease [5]. One possible explanation for the failure of NE inhibitors alone is the challenge with achieving adequate local inhibition in the immediate vicinity of the neutrophils in the lung. Thus, the NE inhibitor may be unable to rapidly quench the extracellular release of high concentrations of NE from a single azurophil granule before they effect tissue damage prior to diffusion to a more dilute concentration that can be effectively inhibited [22]. This tissue injury is a consequence of the quantum proteolysis effect [22]. The failure of NE inhibitors alone, and the promising data for brensocatib in the Phase 2 WILLOW trial [13], suggests that combined inhibition of NE, PR3 and CatG, as well as inhibition prior to extracellular release by the neutrophils, may be required for observation of efficacy in NCFBE.

The thesis that PR3 and CatG are also important regulators of the local inflammatory response is supported by the literature [6, 7, 9, 23] and thus their inhibition may be required to attenuate disease progression in the lung for those with NCFBE. PR3 and CatG, like NE, also process chemokines and stimulate the production of cytokines leading to the activation and mobilization of immune cells to the site of tissue damage [23]. PR3 is not restricted to the azurophilic granules, like NE, and is present in easily mobilizable secretory vesicles [23, 24]. CatG activates metalloproteases and cleaves extracellular matrix proteins, which, in the lung, can contribute to tissue damage. Additionally, in CF bronchial alveolar lavage fluid, surfactant protein A, a peptide that facilities microbial clearance by macrophages, is inhibited to a greater extent by CatG than either NE or PR3, and this can lead to reductions in macrophage phagocytic activity and increased bacterial survival [6]. Furthermore, CatG also inhibits the ability of macrophages to clear apoptotic cells from CF airways, which can lead to increased neutrophil necrosis and an uncontrolled release of proteases into the lung [7]. Finally, in preclinical studies in NE and CatG knock-out mice with *Pseudomonas endobronchitis* infections, examination of bacterial clearance and airway inflammation led to the conclusion that CatG interferes with bacterial clearance and escalates the pulmonary inflammatory response [25]. Thus, all three NSPs may directly contribute to the dysregulation of the local inflammatory environment in the lung in NCFBE [9]. The ongoing Phase 3 ASPEN trial, when complete, may inform on the association of PR3 and CatG with increased exacerbation risk.

## Conclusions

Brensocatib demonstrated dose dependent reductions in blood NE and sputum NSP activity in the WILLOW study. These data suggest a broad anti-inflammatory effect of brensocatib underlying its clinical efficacy observed in NCFBE patients.

## Supplementary Information

**Additional file 1:  Figure S1.** Distribution Analysis of NE Activity in WBCs Collected Over the Trial Duration. NE activity from WBCs is shown in a quantile-quantile plot of measured values versus predicted standard normal distribution values, in a distribution plot, and in a relative frequency histogram plot. The height of the outlined box in panel B indicates the mean value for each arm. Median values for WBC NE are 5772, 4947, 3392 and 4776 ng/mL for placebo, 10 mg brensocatib, 25 mg brensocatib and all arms combined, respectively. **Figure S2.** Distribution Analysis of Sputum NE Activity Collected Over the Trial Duration. NE activity in sputum is shown in a quantile-quantile plot of measured values versus predicted standard normal distribution values, in a distribution plot, and in a relative frequency histogram plot. The bar in panel B indicates the mean NE activity for each arm. Median activity values for each group in the analysis are 3.32, 2.21, 1.50 and 2.30 log (ng/mL) for sputum NE for placebo, 10 mg brensocatib, 25 mg brensocatib and all arms combined, respectively. When converted back from log transformation, the median values are 2089, 162, 32 and 200 ng/mL for sputum NE, respectively. **Figure S3.** Distribution Analysis of Sputum PR3 Activity Collected Over the Trial Duration. PR3 activity in sputum is shown in a quantile-quantile plot of measured values versus predicted standard normal distribution values, in a distribution plot, and in a relative frequency histogram plot. The bar in panel B indicates the mean PR3 activity for each arm. Median activity values are 3.63, 3.60, 3.46, and 3.55 log (ng/mL) for sputum PR3 for placebo, 10 mg brensocatib, 25 mg brensocatib and all arms combined, respectively. When converted back from log transformation, the median values are 4266, 3981, 2884 and 3548 ng/mL for sputum PR3, respectively. **Figure S4.** Distribution Analysis of Sputum CatG Activity Collected Over the Trial Duration. CatG activity in sputum is shown in a quantile-quantile plot of measured values versus predicted standard normal distribution values, in a distribution plot, and in a relative frequency histogram plot. The bar in panel B indicates the mean CatG activity for each arm. Median activity values are 1.29, 0.29, 0.29 and 0.29 log (ng/mL) for sputum PR3 for placebo, 10 mg brensocatib, 25 mg brensocatib and all arms combined, respectively. When converted back from log transformation, the median values are 19, 1.9, 1.9 and 1.9 ng/mL for sputum PR3, respectively.

## Data Availability

All data generated or analysed during this study are included in this published article [and its Additional information files].

## Abbreviations

BQL: Below the quantification limit
CatG: Cathepsin G
CF: Cystic fibrosis
DPP-1: Dipeptidyl peptidase-1
HRP: Horse radish peroxidase
NA: Not applicable
NCFBE: Non-cystic fibrosis bronchiectasis
NE: Neutrophil elastase
NSP: Neutrophil serine protease
NSP-AAP: NSPs-alternative activating protease
PR3: Proteinase 3
SAP: Statistical analysis plan
SEM: Standard error of the mean
WBCs: White blood cells
