# Supplementary material for: Dipeptidyl peptidase-1 inhibition with brensocatib reduces the activity of all major neutrophil serine proteases in patients with bronchiectasis: results from the WILLOW trial
Source: Respir Res. 2023 May 17;24:133. doi: 10.1186/s12931-023-02444-z (PMC10189992; doi:10.1186/s12931-023-02444-z)
Supplement: Supplementary file 1 — Additional file 1: Figure S1. Distribution Analysis of NE Activity in WBCs Collected Over the Trial Duration. NE activity from WBCs is shown in a quantile-quantile plot of measured values versus predicted standard normal distribution values, in a distribution plot, and in a relative frequency histogram plot. The height of the outlined box in panel B indicates the mean value for each arm. Median values for WBC NE are 5772, 4947, 3392 and 4776 ng/mL for placebo, 10 mg brensocatib, 25 mg brensocatib and all arms combined, respectively. Figure S2. Distribution Analysis of Sputum NE Activity Collected Over the Trial Duration. NE activity in sputum is shown in a quantile-quantile plot of measured values versus predicted standard normal distribution values, in a distribution plot, and in a relative frequency histogram plot. The bar in panel B indicates the mean NE activity for each arm. Median activity values for each group in the analysis are 3.32, 2.21, 1.50 and 2.30 log (ng/mL) for sputum NE for placebo, 10 mg brensocatib, 25 mg brensocatib and all arms combined, respectively. When converted back from log transformation, the median values are 2089, 162, 32 and 200 ng/mL for sputum NE, respectively. Figure S3. Distribution Analysis of Sputum PR3 Activity Collected Over the Trial Duration. PR3 activity in sputum is shown in a quantile-quantile plot of measured values versus predicted standard normal distribution values, in a distribution plot, and in a relative frequency histogram plot. The bar in panel B indicates the mean PR3 activity for each arm. Median activity values are 3.63, 3.60, 3.46, and 3.55 log (ng/mL) for sputum PR3 for placebo, 10 mg brensocatib, 25 mg brensocatib and all arms combined, respectively. When converted back from log transformation, the median values are 4266, 3981, 2884 and 3548 ng/mL for sputum PR3, respectively. Figure S4. Distribution Analysis of Sputum CatG Activity Collected Over the Trial Duration. CatG activity in sputum is shown in a qu [file 12931_2023_2444_MOESM1_ESM.docx]

# **ADDITIONAL MATERIALS**

### **Additional Methods**

### WILLOW Statistical Analysis Plan (SAP)

According to the prespecified Statistical Analysis Plan, all subjects who received at least 1 dose of the study drug or placebo, had at least 1 pre-dose and 1 post-dose measurement for NE, PR3, or CatG, and had no major protocol deviations that were considered to impact on the analysis of the PD data, were included in the PD analysis. Missing NSP samples for subjects who were administered scheduled study treatments were considered as non-informative missing samples and were not imputed. No concentration estimates were provided for missing samples. All samples with NSP sputum concentrations that were BLQ were reported as zero. The baseline sputum NSP values represented the average of the sputum NSP values from the subject’s Screening visit and Day 1 (pre-dose) visit as both visits were prior to the initiation of treatment. If one of the Screening or the Day 1 NSP values was below the quantification limit (BQL), then the non-BQL value was used as the Baseline value. If both the baseline and screening samples were BQL, then no change or percentage change from baseline was calculated on treatment.

For the WILLOW trial, there were no primary and only one secondary objective related to NSP biomarkers, which was to evaluate the effect of brensocatib compared with placebo on the concentration of active NE in sputum, as measured by the difference between the pre-treatment concentration and on-treatment concentration and this analysis was previously reported in the primary paper [10]. For that analysis, the measured NE concentration in sputum at each time point was compared to the baseline value for each treatment individually using an ANOVA model. The concentrations were log-transformed assuming a log-normal distribution. BLQ values were excluded from the log-transformation. Treatment was included in the model as fixed either alone or as an interaction treatment over time. Comparison of NE sputum concentrations and change from baseline for each time point was done to the time matched placebo results and derived PD parameters (maximum inhibition or Emax) – to determine the time point where difference from placebo was statistically significant at the 0.05 level. The dose of brensocatib was included as a fixed effect to determine if one or both doses resulted in a significant sputum NE change. The comparison was done either using ANOVA contrasts or PROC MIXED procedure in SAS application for linear mixed effect modeling. The dose for placebo may be imputed = 0. In this case the dose and NE concentrations may not be log-transformed to mitigate possibility of change from baseline or measured concentration values equal to 0.

Statistical analyses

Statistical analyses were performed using GraphPad Prism (version 9.2.0, La Jolla, CA). Two-way analysis of variance (ANOVA) utilized an alpha value set at 0.05 to fit a full model of column and row interaction effects, and then a Dunnett’s multiple comparison test was performed. Statistical significance was considered for P values < 0.05. The Normality and Lognormality of distributions were determined using the Anderson-Darling test with significance level (alpha) set at 0.05. Simple linear regression with a best-fit model was performed using GraphPad Prism; Pearson correlation coefficients (r) were calculated using the same software. The P value of Pearson correlation was also calculated, which represents the probability that one would have found the current result if the correlation coefficient were in fact zero (null hypothesis). If P value < 0.05, the correlation coefficient is considered statistically significant.

**Additional Results and Discussion**

### Distribution Analysis of WBC NE Activities

Blood was collected from subjects at baseline, 4, 12 and 24 weeks on treatment, and then at 28 weeks, 4 weeks after the end of treatment. The extracts from the WBC samples were evaluated for NE activity. A distribution test was conducted to determine whether the complete data set of WBC NE activity was defined by normal or lognormal distributions.

WBC NE data values for each of the three arms (placebo, 10 mg brensocatib, and 25 mg brensocatib) collected from all the subject visits were distributed normally **(Figure S1A)**. The individual data points representing each subject’s blood NE activity at each visit also followed a normal distribution pattern in the placebo arm, but the NE levels shifted lower in the brensocatib arms, especially for the 25 mg dose, compared to placebo **(Figure S1B and C)**.

### Distribution Analysis of Sputum NSPs

A distribution test was also conducted on the active NSP levels in sputum. Sputum NE levels for each of the three arms followed a lognormal distribution pattern. Therefore, a log transformation was performed on the sputum dataset and this data set was used for subsequent analysis. The log transformed data indeed was more closely aligned with a normal distribution **(Figure S2A)**. After log transformation, the individual data points representing each subject’s sputum NE level at each visit aligned with a typical normal distribution pattern in the placebo arm (excluding the data points with values below the quantitation limit (BQL)) **(Figure S2B and S2C)**. The median NE levels in the brensocatib arms, especially for the 25 mg dose, were reduced compared to placebo **(Figure S2B)**.

Sputum PR3 for each of the three arms also followed a lognormal distribution and the log transformed data closely aligned with a normal distribution **(Figure S3A)**. After log transformation, the individual PR3 data values demonstrated a relatively typical normal distribution pattern in the placebo and brensocatib arms **(Figure S3B and S3C)**. The median PR3 level in the 25 mg brensocatib arm was reduced compared to placebo **(Figure S3C)**.

Sputum CatG for each of the three arms followed a very similar pattern to sputum NE, and the log converted data was consistent with a normal distribution **(Figure S4A)**. After log transformation, the data followed a typical normal distribution pattern in the placebo arm (excluding the data points with BQL values) **(Figure S4B and S4C)**. We also observed that there were lower median CatG levels in the brensocatib arms, especially for the 25 mg dose, compared to placebo **(Figure S4B and S4C)**.

## **Additional Figures:**

Figure S1. Distribution Analysis of NE Activity in WBCs Collected Over the Trial Duration.

NE activity from WBCs is shown in a quantile-quantile plot of measured values versus predicted standard normal distribution values (A), in a distribution plot (B), and in a relative frequency histogram plot (C). The height of the outlined box in panel B indicates the mean value for each arm. Median values for WBC NE are 5772, 4947, 3392 and 4776 ng/mL for placebo, 10 mg brensocatib, 25 mg brensocatib and all arms combined, respectively.

Figure S2. Distribution Analysis of Sputum NE Activity Collected Over the Trial Duration.

NE activity in sputum is shown in a quantile-quantile plot of measured values versus predicted standard normal distribution values (A), in a distribution plot (B), and in a relative frequency histogram plot (C). The bar in panel B indicates the median NE activity for each arm. Mean activity values for each group in the analysis are 3.32, 2.21, 1.50 and 2.30 log(ng/mL) for sputum NE for placebo, 10 mg brensocatib, 25 mg brensocatib and all arms combined, respectively. When converted back from log transformation, the median values are 2089, 162, 32 and 200 ng/mL for sputum NE, respectively.

Figure S3. Distribution Analysis of Sputum PR3 Activity Collected Over the Trial Duration.

PR3 activity in sputum is shown in a quantile-quantile plot of measured values versus predicted standard normal distribution values (A), in a distribution plot (B), and in a relative frequency histogram plot (C). The bar in panel B indicates the median PR3 activity for each arm. Mean activity values are 3.63, 3.60, 3.46, and 3.55 log(ng/mL) for sputum PR3 for placebo, 10 mg brensocatib, 25 mg brensocatib and all arms combined, respectively. When converted back from log transformation, the median values are 4266, 3981, 2884 and 3548 ng/mL for sputum PR3, respectively.

Figure S4. Distribution Analysis of Sputum CatG Activity Collected Over the Trial Duration.

CatG activity in sputum is shown in a quantile-quantile plot of measured values versus predicted standard normal distribution values (A), in a distribution plot (B), and in a relative frequency histogram plot (C). The bar in panel B indicates the median CatG activity for each arm. Mean activity values are 1.29, 0.29, 0.29 and 0.29 log(ng/mL) for sputum PR3 for placebo, 10 mg brensocatib, 25 mg brensocatib and all arms combined, respectively. When converted back from log transformation, the median values are 19, 1.9, 1.9 and 1.9 ng/mL for sputum PR3, respectively.
